# Degradation and transformation mechanisms of numbing substances: Hydroxyl-α-sanshool & hydroxyl-β-sanshool from *Zanthoxylum bungeanum* exposed to acid environment

**DOI:** 10.1016/j.fochx.2022.100342

**Published:** 2022-05-21

**Authors:** Jingjing Luo, Xiaoyan Hou, Shanshan Li, Qingying Luo, Hejun Wu, Guanghui Shen, Xuequan Gu, Xiaoyan Mo, Zhiqing Zhang

**Affiliations:** a College of Food Science, Sichuan Agricultural University, Ya’an 625014, China; b Sichuan Wufeng Lihong Food Co Ltd, Hanyuan 625300, China

**Keywords:** *Zanthoxylum bungeanum*, Acidification, Numbing substances, Degradation mechanism

## Abstract

In present work, *Zanthoxylum bungeanum* meal (ZBM) used as experimental material, the stability of typical alkylamides (hydroxyl-α-sanshool and hydroxyl-β-sanshool) in ZBM under different acidification conditions was investigated, in order to reveal degradation or transformation mechanism of numbing substances from *Z. bungeanum* exposed to acid environment and its transform direction. The alkylamides content of ZBM was detected by using HPLC after different conditions of acidification. The results indicated that hydroxyl-α-sanshool and hydroxyl-β-sanshool under the concentration of hydrochloric acid is 14% decreased by 80% after only 0.5 h. Moreover, some of the components undergo isomerization and addition reactions in the process of acidification, the products of isomerization are hydroxyl-ε-sanshool and (1Z,2E,4E,8E,10E)-N-(2-hydroxy-2-methylpropyl)dodeca-2,4,8,10-tetraenimidic acid; and the product of the addition reaction is (2E,6E,8E,10E)-1-chloro-1-(2-hydroxy-2-methylpropyl)amino)dodeca-2,6,8,10-tetraen-1-ol, which indicated that acid environment has greatly changed the numbing substances in *Z. bungeanum* and its products.

## Highlights

• Isomerization and addition reactions of sanshools reduced the numbing degree for the first time.
• Acidic environment promotes isomerization and addition reactions of numbing substances.
• The degradation rates of hydroxyl-α-sanshool was 75.36%.

## 1 Introduction

Chinese prickly ash (*Zanthoxylum bungeanum*) is an important traditional Chinese flavouring and functional food in East and Southeast Asians (Lu et al., 2020, Yang et al., 2021, Wang et al., 2020). The pericarp of *Z. bungeanum* is rich in numbness flavor produced by long chain unsaturated alkylamides, and aroma components composed of volatile oil (Rashed Marwan et al., 2021, Chen et al., 2018). *Z. bungeanum* fruit is widely used as a seasoning because of its unique numbness and aroma. Fresh fruit is not easy to preserve, more than 30% is processed into *Z. bungeanum* oil, which is not only conducive to the preservation of its original aroma and numbness, but also greatly reduce the loss of effective ingredients in the storage and sales process (Ji et al., 2019). *Zanthoxylum bungeanum* meal (ZBM) is the main by-product produced in the production of *Z. bungeanum* oil. More literatures have proved that ZBM contains high nutritional values, such as protein, oil and dietary fiber, etc (Jiang et al., 2014, Wang et al., 2017, Jiang et al., 2020). Compared with other agricultural by-products, ZBM has high antibacterial, anti-inflammatory and antioxidant activities, which is a potential good source of food additives (Wu et al., 2016). In addition to the above nutrients, ZBM also contains a lot of numbing substances (Wu et al., 2020). On the one hand, the numbing flavor of *Z. bungeanum* was weakened during storage; On the other hand, except in Southwest China, people in most parts of China do not adapt to the strong numbing flavor in food, which lead to the restriction of food development and utilization of *Z. bungeanum*. Therefore, it is necessary to deeply understand the degradation and transformation mechanisms of numbing substance in order to develop pepper food categories suitable for different regions.

In recent years, more researchers have proved that the main numbing substances of *Z. bungeanum* are alkylamides, such as hydroxyl-α-sanshool, hydroxyl-β-sanshool etc. (Huang et al., 2012, Wang et al., 2017, Gong et al., 2021). A limited number of literatures have showed that the isomerization or degradation of hydroxyl-α-sanshool is closely related to environmental factors. Yang (2008) reported that hydroxyl-α-sanshool degradation completely after 4 h of UV irradiation. Li et al. (2021) isolated and identified strains *L. paracasei* and *L. acidipiscis* for the degradation of numbing in ZBM. The results showed that the degradation rate of the two kinds of *Lactobacillus* to numbing substances reached 51.86%. Ichiro et al. (1982) prepared hydroxyl-β-sanshool by iodine-catalyzed hydroxyl-α-sanshool. Among environmental factors, oxygen, temperature and pH are most important factors affecting the structure and content of numbing substances (Schweiggert et al., 2006, Topuz and Ozdemir, 2004). The alkylamides in *Z. bungeanum* are easily oxidized due to the presence of three conjugated double bonds (Bryant & Mezine, 1999). Cheng et al. (2021) investigated the impact of different storage conditions on the qualities of dried *Z. armatum*, including color, numbness, and aroma, for seven months to identify a suitable storage method. The result showed that the optimal way to preserve dried *Z. armatum* was non-light packaging at −18 °C, and room temperature and light can obviously reduce the content of numbing substances. Hydrochloric acid has a strong reducibility. It can add *alkene* double bond, alkylamides into other effective substances, as well as increasing the solubility of alkylamides in water (Gao et al., 2019). However, there are few reports on the degradation and transformation mechanism of numbing substances.

In this study, the stability of typical hydroxyl-α-sanshool and hydroxyl-β-sanshool in ZBM under different acidification conditions was investigated. The content changes of hydroxyl-α-sanshool and hydroxyl-β-sanshool were determined, and the degradation and transformation mechanism of numbing substances in acidic environment was analyzed. Results indicated that hydroxyl-α-sanshool and hydroxyl-β-sanshool are extremely unstable in acidic environment and are prone to isomerization and addition reactions, which provided reference for numbing substances change of *Z. bungeanum* and its products during storage and processing, meanwhile, promoted the comprehensive utilization of *Z. bungeanum* and its products.

## 2 Materials and methods

### 2.1 Chemicals and experimental material

Methanol (chromatographic grade), acetonitrile (chromatographic grade), hydrochloric acid (HCl, 11.6 mol/L) and sodium hydroxide (NaOH, 1 mol/L) were purchased from Ke Long Chemical Reagent Co., Ltd (Chengdu, China). The standard hydroxy-α-sanshool and hydroxy-β-sanshool were purchased from Chengdu RefMedic Biotech Co., Ltd. (Chengdu, China, purity ≥ 99.00%). The standard linalool was purchased from Chengdu Chroma-Biotechnology Co., Ltd. (Chengdu, China, purity ≥ 98.00%).

ZBM was provided by Sichuan Wufeng Lihong Food Co., Ltd (Hanyuan, China).

### 2.2 Pretreatment of ZBM

The ZBM was smashed by a universal high-speed pulverizer (FW-100, Beijing Zhongxing Weiye Instrument Co., Ltd., China), and sieve it with a particle size of 250 μm to get ZBM powder.

### 2.3 Determination of initial numbing substances of ZBM

10.0 g of ZBM powder was dissolved in 20 mL ultra-pure water with stirring constantly. The mixture was incubated in water bath (HH-2, Jiangsu Ronghua Instrument Manufacturing Co. Lid., China) at 35 °C for 30 min. Then, ultrasonic (KH-300DE, Kunshan Hechuang Ultrasonic Instrument Co., Lid., China) assisted extraction (35 min) was conducted with 70% methanol added in a solid–liquid ratio of 1:10. After filtration, the crude extract of numbing substances was obtained. Separation and detection of numbing substances were carried out on the HPLC system (Agilent, USA), the chromatographic separation was performed on an Agilent Eclipse XDB-C18 column (4.6 mm × 150 mm, 5 μm) and DAD (Agilent, USA) detector. The mobile phase was water (A) – acetonitrile (B), the elution gradient was 0 min, 65% A; 5–10 min, 65–60% A; 10–55 min, 60–10% A; 55–60 min, 10–65% A. The detection wavelength was 268 nm.

### 2.4 Sensory evaluation of initial numbing degree of ZBM

Sensory evaluation is used to analyze the numbing degree (G value) according Scoville pungency unit (SPU) (Zhao et al., 2020).

#### 2.4.1 Preparation of diluent of numbing substance

A quantitative numbing substance extract was taken and purified water was applied to a constant volume of 50 mL until the evaluator identified the minimum sample volume for numbing substance as the minimum threshold of numbing substance. Finally, the corresponding Scoville pungency unit (SPU) are converted according to the minimum dilution multiple under the threshold value.

#### 2.4.2 Evaluation method

During the evaluation process, the sample cups were randomly coded, and the evaluators also randomly sensed (the evaluation team should meet the basic requirements of GB/T 16291.1-2012 for preferred evaluators). Meanwhile, the same number of times for each sample was evaluated should be guaranteed.

### 2.5 Degradation rate of numbing substances

In order to intuitively see the influence of hydrochloric acid on numbing substances of ZBM, the degradation rate was expressed by the content changes of hydroxyl-α-sanshool and hydroxyl-β-sanshool in the sample, and the degradation rate formula was as follows:

$$\mathrm{Degradation}\;rate\left(\%\right)=\frac{H1-H2}{H1}$$

In the formula, H1 is the initial content/% of numbing substances in ZBM; H2 is the residual content/% of numbing substances in ZBM after degradation.

### 2.6 Degradation processing of hydrochloric acid on numbing substances of ZBM

Add 20 mL 6% hydrochloric acid and 10 g of ZBM powder into 250 mL conical flask, shake well, incubate in water bath (35 °C) for 30 min, and then neutralize with 1 mol/L sodium hydroxide standard solution until pH 7, At the same time, HPLC was used to quantitatively analyze the content of numbing substances in ZBM, Sensory evaluation was carried out to determine the degree of numbing.

### 2.7 Analysis of optimum degradation conditions

The four most important factors which were set as follows: raw material size (0.425 mm, 0.300 mm 0.250 mm, 0.180 mm, 0.150 mm, 0.125 mm); hydrochloric acid concentration (6%, 8%, 10%, 12%, 14%, 16%); reaction temperature (20 °C, 25 °C, 30 °C, 35 °C, 40 °C, 45 °C); reaction time 15 min, 20 min, 25 min, 30 min, 35 min, 40 min). The average degradation rate of each trial as well as the average numbing degree (G value) for individual factors at different level was calculated and used to evaluate the efficiency and optimize experimental conditions of degradation.

### 2.8 Analysis of degradation mechanism of numbing substances

#### 2.8.1 Effect of hydrochloric acid on the structure of hydroxyl-α-sanshool and hydroxyl-β-sanshool

The hydroxyl-α-sanshool standard solution was divided into five parts numbered AH1-AH5. Then 10 mL hydrochloric acid with different concentrations (0%, 4%, 8%, 12%, 14%) was added respectively. The solution was incubated in water bath at 35 °C for 30 min.

The degradation treatment of hydroxy-β-sanshool is the same as above, hydrochloric acid with different concentrations (0%, 4%, 8%, 12%, 14%) was added.

Analysis structural changes of hydroxyl-α-sanshool and hydroxyl-β-sanshool HPLC, FT-IR and LC-MS were used to.

#### 2.8.2 Analysis for structural changes of two sanshools by FT-IR

The resolution of the instrument was 4 cm^−1^, and the scanning range was 4000∼400 cm^−1^. The air was used as the background. The instrument was scanned for 32 times, and each sample was measured in parallel for 3 times. All measured spectra were subtracted from the air background spectrum.

#### 2.8.3 Analysis for structural changes of two sanshools by LC-MS

HPLC was performed on Hypersil Gold C_18_ column (2.1 × 100 mm, 1.9 μm). Mobile phase: acetonitrile: water (50:50); Temperature: 35 °C; Flow rate: 0.3 mL/min; Sample size: 5 μL. Mass spectral parameters: Ion source type: ESI; Scanning mode: positive ion mode; Detection method: multiple response detection (MRM); Dry temperature: 325 °C; Dry gas flow: 6 L/min: atomizer pressure: 35 PSI; Scabbard temperature: 350 °C; Sheath gas flow: 10 L/min; Capillary voltage: 3500 V; Nozzle voltage: 0 V. The standard spectrum library was NIST 11.1.

### 2.9 Statistical analysis

Graphical presentation and statistical analysis of the data were carried out by MATLAB 2014 was applied for data and Origin 9.0.

## 3 Results and discussion

### 3.1 Determination of initial numbing substance content and numbing degree of ZBM

The curve equations of hydroxyl-α-sanshool and hydroxyl-β-sanshool are respectively as follows: Y_1_ = 0.9907X-6.7354, *r*_1_ = 0.9985; Y_2_ = 1.2946X + 0.0504, *r*_2_ = 0.9981.

According to the peak area and standard curve, the initial content of numbing substances in ZBM were calculated, and sensory evaluation (numbing degree) was conducted on the extracted numbing substances. The content of hydroxyl-α-sanshool (CS1) in the three groups of ZBM is more than 98 ± 0.87 µg·mL^−1^, and the content of hydroxyl-β-sanshool (CS2) is 8.17 ± 1.01 μg·mL^−1^. The initial numbing degree (G) of ZBM is 5.22 ± 0.66 according Scoville's index (SPU) method (Zhao et al., 2020) (Table 1).

**Table 1:** Initial numbing substance content and numbing degree of ZBM.

Number of samples	AS1(mAU)	CS1(μg/mL)	AS2(mAU)	CS2(μg/mL)	G
A-1	90.8226 ± 0.91	98.47 ± 0.86	10.5798 ± 1.08	8.17 ± 1.12	5.13 ± 0.33
A-2	91.0045 ± 0.53	98.59 ± 0.67	10.6902 ± 0.77	8.18 ± 0.89	5.09 ± 0.59
A-3	90.7633 ± 1.12	98.56 ± 1.08	10.6988 ± 0.86	8.17 ± 1.03	5.44 ± 1.06

Note: AS1: peak area of hydroxyl-α-sanshool; CS1: content of hydroxyl-α-sanshool; AS2: peak area of hydroxyl-β-sanshool; CS2: content of hydroxyl-β-sanshool; G: numbing degree.

### 3.2 Degradation effect of numbing substance with hydrochloric acid

In order to verify the feasibility of degrading numbing substances by hydrochloric acid, the of the treatment effects on numbing substances of hydrochloric acid, sodium hydroxide and hydrogen peroxide were analyzed primarily. Hydrochloric acid has the strongest destructive effect on numbing substances at the same concentration, with the degradation rate of hydroxyl-α-sanshool (DS1) of 31.83 ± 0.93% and the degradation rate of hydroxyl-β-sanshool (DS2) of 21.21 ± 0.56%, and G value of 4.01 ± 0.95, which is one grade lower than the initial degree of numbing substances. The second is hydrogen peroxide, whose DS1 value is 26.59 ± 0.66%, DS2 value is 19.13 ± 0.67%, and G value is 4.56 ± 0.61. Sodium hydroxide had little effect on the two kinds of sanshool in ZBM (Table 2).

**Table 2:** Influence results of different reagents on numbing substances.

Degrade reagents	AS1(mAU)	CS1(μg/mL)	DS1(%)	AS2(mAU)	CS2(μg/mL)	DS2(%)	G
NaOH	87.8506 ± 0.66	95.47 ± 0.57	3.08 ± 0.31	9.3795 ± 0.94	7.21 ± 0.67	12.08 ± 0.55	5.03 ± 0.84
H_2_O_2_	64.9142 ± 1.91	72.31 ± 0.89	26.59 ± 0.66	8.6313 ± 1.44	6.63 ± 0.82	19.13 ± 0.67	4.56 ± 0.61
HCl	59.8004 ± 1.21	67.16 ± 1.09	31.83 ± 0.93	8.411 ± 1.02	6.46 ± 0.78	21.21 ± 0.56	4.01 ± 0.95

Note: DS1: Degradation rate of hydroxyl-α-sanshool; DS2: Degradation rate of hydroxyl-β-sanshool.

### 3.3 Analysis of optimum degradation conditions through orthogonal design experiment

The orthogonal array design optimization of the degradation conditions was based on the maximum degradation rate of the sample. All parameters were tested in a wider range prior to orthogonal array design optimization. This helped narrowing down the ranges of the parameters tested.

**Fig. 1:**
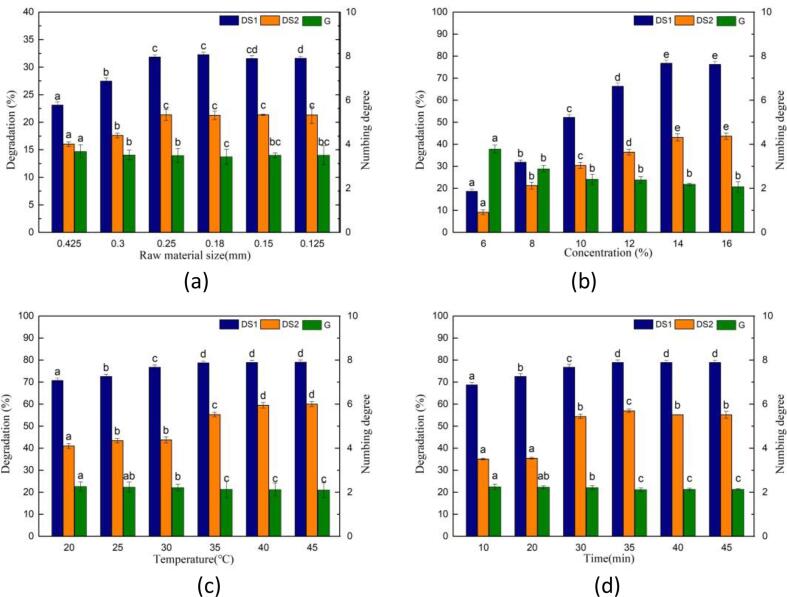
Effect of material size (a), hydrochloric acid concentration (b), temperature (c), and time (d) on the degradation rate of numbing substances.

In Fig. 1 (a), 10 g of ZBM with different material sizes were weighed and degraded under the conditions of hydrochloric acid concentration of 6%, reaction time of 40 min at 30 °C, according to the solid–liquid ratio of 1:2. With the increase of material size, the degradation rate increased firstly and then decreased, and the difference was not significant (*P* > 0.05). When the material size was between 0.250 mm and 0.180 mm, the numbing degree of ZBM decreased first and then stabilized. When the material size reached 0.180 mm, the degradation rate of numbing substances reached the maximum, the DS1 was 32.25 ± 1.03%, DS2 was 21.25 ± 0.75%, and the numbing degree tended to be stable, the G was 3.5 ± 0.77. The reason for this phenomenon is that the pulverization of ZBM is not sufficient, and the surface area of numbing substances in contact with the solvent is relatively small, leading to a relatively low degradation rate. When ZBM was crushed too fine, the adsorption of particles in the degradation process of numbing substances was strengthened, and the solvent was not easy to contact with numbing substances, leading to a decrease in the degradation rate (An & Wang, 2021). Therefore, the material size of raw material is 0.180 mm.

The effects of different hydrochloric acid concentrations on degradation rate and numbing grade were discussed under the condition of the same material size, time and temperature of ZBM. In Fig. 1 (b), the DS1 value significantly increased with the increase of hydrochloric acid concentration (*P* < 0.05), while the DS2 value showed a trend of slowly increase (*P* > 0.05). When the concentration reached 14%, the degradation rate reached the maximum (with DS1 of 71.95 ± 1.07% and DS2 of 40.69 ± 0.75%). The G value dropped from 5.06 ± 0.77 to 3.12 ± 0.91. The structure of hydroxyl-α-sanshool contains two conjugated double bonds and is prone to decomposition, while the structure of hydroxyl-β-sanshool is more stable than that of hydroxyl-α-sanshool (Ichiro et al., 1982, Kristin and Diana, 2009). Therefore, 14% hydrochloric acid concentration is the best.

Under the conditions of material size of 0.180 mm, concentration of 14% and time of 40 min, the effects of different temperatures on the degradation rate were investigated. In Fig. 1 (c). with the increase of temperature, the value of DS1 increases gradually and obtained a saturated value at the end. While the value of DS2 firstly keeps balance and then increases slowly until it reaches saturation. When the temperature is low, the structure of hydroxyl-β-sanshool is stable and its molecular structure is not active, while the structure of hydroxyl-α-sanshool is unstable (Sugai et al., 2005, Albin and Simons, 2010), with the increase of temperature, the molecular thermal motion rate is accelerated. In the same time, the value of DS1 increases with the increase of temperature, while the value of DS2 does not significantly increase. However, when the temperature was higher and the temperature continued to rise, the structure of hydroxyl-β-sanshool became active under the influence of high temperature and underwent transformation or degradation. Therefore, in the same time, the value of DS1 in the system tended to be saturated, while the value of DS2 showed a significant increase. Combined with the change of numbing grade, the optimum temperature is 35 °C.

Under the conditions of material size 0.180 mm, concentration 14% and temperature 35 °C, the effects of different time on the degradation rate of ZBM were investigated. In Fig. 1 (d), with the increase of time, the degradation rate of sanshool gradually increase and then come closer to balance, and there was no significant difference from 35 min to 45 min (*P* > 0.05). With the extension of time, hydrochloric acid will degrade not only numbing substances, but also other compounds of ZBM. Therefore, the solution reaches saturation after 35 min, and the degradation rate will not increase (Wu et al., 2012). Therefore, 35 min is chosen as the best reaction time.

The orthogonal experiment results as seen in Table 3. DS1 as the evaluation index, the primary and secondary order of the four factors affecting the degradation rate is concentration > material size > temperature > time, and the optimal extraction condition is A_3_B_3_C_3_D_2_. DS2 as the evaluation index, the primary and secondary order of influencing factors is concentration > material size > temperature > time, and the optimal process combination is A_3_B_3_C_3_D_2_. G as the evaluation index, the primary and secondary order of influencing factors is concentration > material size > temperature = time, and the optimal process combination is A_3_B_3_C_2_D_3_. In conclusion, the optimal process combination is A_3_B_3_C_2_D_3_. Under this condition, the degradation rate of hydroxyl-α-sanshool (DS1) is 75.56 ± 1.04%, the degradation rate of hydroxyl-β-sanshool (DS2) is 42.69 ± 0.73%, and the degree of numbing (G) decreased from 5.22 ± 0.66 to 2.17 ± 0.91.

**Table 3:** Orthogonal array design and result for the optimization of degrade conditions.

NO	Factor				DS1(%)	DS2(%)	G
	A	B	C	D			
1	1	1	1	1	62.420 ± 1.02	33.688 ± 0.66	2.31 ± 0.54
2	1	2	2	2	71.896 ± 0.55	36.609 ± 0.83	2.25 ± 1.01
3	1	3	3	3	75.160 ± 0.42	39.382 ± 0.71	2.22 ± 0.96
4	2	1	2	3	65.391 ± 0.75	35.054 ± 1.11	2.28 ± 0.43
5	2	2	3	1	70.565 ± 0.65	38.421 ± 1.04	2.26 ± 0.64
6	2	3	1	2	73.263 ± 0.37	40.812 ± 0.94	2.24 ± 1.05
7	3	1	3	2	69.046 ± 1.04	38.621 ± 0.56	2.27 ± 0.56
8	3	2	1	3	72.854 ± 1.12	40.915 ± 0.76	2.24 ± 0.72
9	3	3	2	1	75.559 ± 0.93	42.689 ± 0.53	2.21 ± 0.95
DS1	k_1_	69.825	65.619	69.512	69.515		
	k_2_	69.739	71.772	70.949	71.402		
	k_3_	72.486	74.661	71.59	71.135		
	R	2.747	9.042	2.078	1.887		
Primary and secondary factors:B > A > C > D optimal portfolio:A_3_B_3_C_3_D_2_							
DS2	k_1_	36.560	35.787	38.472	38.266		
	k_2_	38.096	38.649	38.117	38.681		
	k_3_	40.742	40.961	38.808	38.450		
	R	4.182	5.174	0.691	0.414		
Primary and secondary factors:B > A > C > D optimal portfolio:A_3_B_3_C_3_D_2_							
G	k_1_	2.26	2.287	2.263	2.26		
	k_2_	2.26	2.25	2.247	2.253		
	k_3_	2.24	2.223	2.25	2.247		
	R	0.02	0.06	0.013	0.013		
	Primary and secondary factors:B > A > C = D optimal portfolio:A_3_B_3_C_2_D_3_						

### 3.4 Investigation of degradation mechanisms of numbing substances

#### 3.4.1 The analysis results of HPLC

**Fig. 2:**
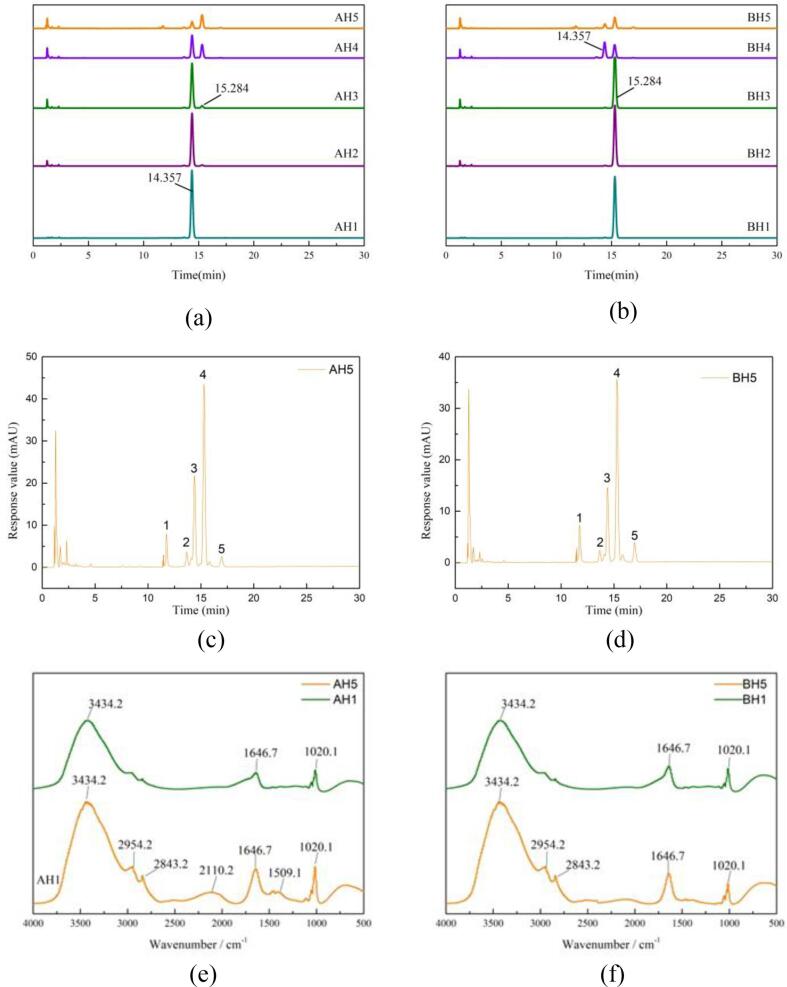
Analysis of compounds from AH1-AH5 (a) and BH1-BH5 (b) by HPLC; Effect of 14% hydrochloric acid on the chromatographic peaks of hydroxyl-α-sanshool (c) and hydroxyl-β-sanshool (d) by HPLC. The infrared fingerprints of AH5 (e) and BH5 (f).

In Fig. 2, the concentration of hydrochloric acid was less than 8%, the peak areas of hydroxyl-α-sanshool and hydroxyl-β-sanshool decreased with the increase of the concentration. Meanwhile, the peak of hydroxyl-α-sanshool changed from the original single to two peaks (Fig. 2 (a)), while the peak of hydroxyl-β-sanshool did not change. When the concentration of hydrochloric acid increased to 12%, the peak area of the two kinds of sanshool decreased sharply. At this time, the peak of hydroxyl-β-sanshool also changed from a single peak to two peaks (Fig. 2 (b)). When the concentration of hydrochloric acid was increased to 14%, the two kinds of sanshool decreased by 80% only after 0.5 h, and at the same time, the peaks changed from 2 to 5 (Fig. 2 (c)+(d)). The reason is that when the concentration is low, hydroxyl-α-sanshool has *cis* double bond and is prone to oxidation, isomerization, polymerization and other reactions (Yang, 2008, Iseli et al., 2007), while hydroxyl-β-sanshool does not have *cis* double bond and has a relatively stable structure, and low concentration of hydrochloric acid does not cause the isomerization of hydroxyl-β-sanshool. When the concentration of hydrochloric acid increases, although hydroxyl-β-sanshool is more stable than hydroxyl-α-sanshool, due to its highly unsaturated nature (Kolodiazhna & Kolodiazhnyi, 2018), it also eventually isomerizes.

According to the peak area (Fig. 2) and standard curve equation, the contents of hydroxyl-α-sanshool and hydroxyl-β-sanshool at different concentrations were calculated and fitted according to the first-order reaction rate equation. The simulation equations of content (S) and concentration (C) were obtained: S_1_ = 274.93–54.68e^0.1067c1^, S_2_ = 156.175–5.323e^0.2281C2^. The fitting degree was good. From this equation, the degradation rate of sanshools in different acidic environments can be estimated.

In Fig. 2 (c) and (d), when the concentration of hydrochloric acid increased to 14%, the changes of AH5 and BH5 were basically the same, changing from single peak to five peaks, and the retention time of from 1 to 5 was 12.857 min, 13.667 min, 14.357 min, 15.284 min and 17.018 min, respectively. According to the retention time, it was preliminarily judged that the third peak was hydroxyl-α-sanshool, the fourth peak was hydroxyl-β-sanshool, and the substances in the first, second and fifth peaks needed to be further determined.

#### 3.4.2 The analysis results of FT-IR

In Fig. 2 ((e)+(f)), the spectrum obtained by infrared scanning is mainly divided into two bands: 3500∼2800 cm^−1^ and 2200∼1000 cm^−1^. Among them, the strong wide peak near 3434.2 cm^−1^ is the absorption peak caused by N=O stretching vibration of protein and O=H contraction vibration of water molecule. The absorption peaks near 2954.2 cm^−1^ and 2843.2 cm^−1^ were induced by *cis* and *trans* stretching vibrations of methylene, respectively. 2110.2 cm^−1^ is the absorption peak caused by the stretching vibration of carbonyl group C=O in aldehydes. The C=C absorption peak of ketone is near 1646.7 cm^−1^. The characteristic absorption peak of amide I band is near 1509.1 cm^−1^. 1020.1 cm^−1^ is the absorption peak caused by bending vibration of C—H bond (Qi and Wu, 2014, Ke et al., 2017).

In Fig. 2, after adding 14% hydrochloric acid, the absorption intensity of AH5 (Fig. 2 (e)) was weakened at 3434.2 cm^−1^, 2954.2 cm^−1^, 2843.2 cm^−1^, 1646.7 cm^−1^ and 1020.1 cm^−1^, and the absorption peak disappeared at 2110.2 cm^−1^ and 1509.1 cm^−1^. The absorption intensity of BH5 (Fig. 2 (f)) at 3434.2 cm^−1^, 2954.2 cm^−1^, and 2843.2 cm^−1^ was significantly weakened, which was because hydrochloric acid degraded most of the sanshool, and at the same time, a small amount of sanshool was isomerized (Zhao et al., 2013, Yan et al., 2017). The FT-IR of the two kinds of sanshool and hydrochloric acid still contain the characteristic bands of amides, so it is speculated that the products may have amides analogues.

#### 3.4.3 The analysis results of LC-MS

**Fig. 3:**
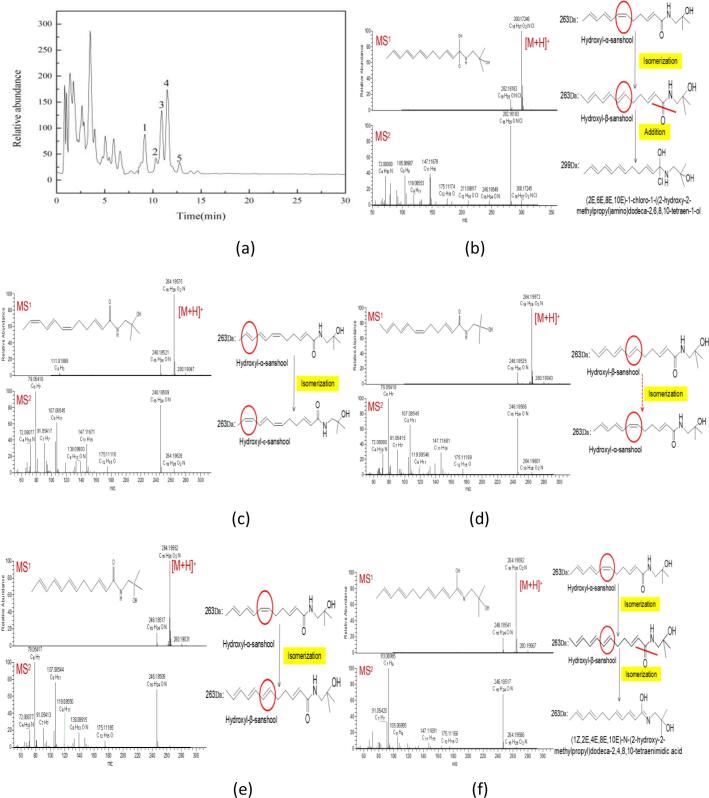
The total ion chromatograms of AH5 (a); Mass spectrometry, structural formula and transformation path of peak 1 (b), peak 2 (c); peak 3 (d); peak 4 (e), peak 5 (f).

The results of HPLC analysis showed that the effect of 14% hydrochloric acid on hydroxyl-α-sanshool and hydroxyl-β-sanshool was basically the same (Fig. 2 (c)+(d)). Therefore, the structural changes of hydroxyl-α-sanshool were only analyzed in this paper. Fig. 3 (a) shows the total ion flow diagram after the action of hydroxyl-α-sanshool with 14% hydrochloric acid. The peak of 0∼7 min is the solvent peak, the target peak 1∼5, and the retention time is 9.1921 min, 10.2750 min, 10.9454 min, 11.5320 min, 12.8664 min, respectively.

Through the analysis of the ion flow diagram extracted from the sample and the information of the first and second order fragments with molecular weights between 50∼600, each chromatographic peak in the sample is given by the ESI (+) mass spectrometry [M+H]^+^ ions and *m*/*z*, and the ions can be determined as hydrogenated ions, that is, the molecular weight of the compound can be obtained: the molecular weight of peak 1 is 299 Da, the molecular weight of peak 2∼5 was 263 Da. According to the large difference in molecular weight, it was speculated that peak 1 might be an adjunct of sanshools and chloride ion, while peak 2∼5 was an analogue of sanshools, which were isomers of each other. According to the secondary spectrogram information of the high resolution mass spectrometry, the composition and isotope distribution of the compounds were calculated. Combined with the data analysis in the references (Kumar et al., 2014, Etsuko et al., 2014, Bhatt et al., 2017, Ji et al., 2019), the material structure and transformation path of peaks 1∼5 were obtained (Fig. 3(b)∼(f)), and the specific analysis results are as follows:

The mass spectrogram (MS^1^) of LC-MS was the molecular peak, and the relative molecular mass of the substance could be seen from Fig. 3(b). The maximum abundance [M+H]^+^ ion was 300.1. According to the MS^2^ mass spectrogram, the ion was determined to be a hydrogenation ion, and the excimer ion information of the substance was finally obtained: The molecular weight of the peak 1 compound was 299 Da. The molecular weight of the unknown compound 1 is 36 larger than that of hydroxyl-α-sanshool, which is exactly the mass of one Cl atom plus one H atom. Combined with the properties of sanshools and the conditions of the whole reaction system, it is speculated that the unknown compound 1 is the addition of sanshools and hydrochloric acid. According to the small molecular structure of material fragments given by MS^2^ mass spectrometry, and through software analysis and literature references (Etsuko et al., 2014). The substances peak 1 was::(2E,6E,8E,10E)-1-chloro-1-((2-hydroxy-2-methylpropyl)amino)dodeca-2,6,8,10-tetraen-1-ol. By analyzing its structural formula and molecular weight changes, the transformation path of peak 1 was predicted (Fig. 3(b)): The first step: the homeopathic double bond of hydroxyl-α-sanshool (M = 263 Da) isomerized to produce hydroxyl-β-sanshool (M = 263 Da). The second step: hydroxyl-β-sanshool reacts with hydrochloric acid to produce (2E,6E,8E,10E)-1-chloro-1-((2-hydroxy-2-methylpropyl)amino)dodeca-2,6,8, 10-Tetraen-1-ol (M = 299 Da).

The maximum abundance [M+H]^+^ ion given by MS^1^ mass spectrometry (Fig. 3(c)) is 264.1, indicated the molecular weight of peak 2 compound was 263 Da. As the chromatographic peaks of the peak 3 and peak 4 have been preliminary determined as hydroxyl-α-sanshool and hydroxyl-β-sanshool, the isomers of the peak 2 are mainly investigated here. According to reference (Chen et al., 2014), the molecular weight of hydroxyl-ε-sanshool is 263 Da, and the retention time of hydroxyl-ε-sanshool is second only to hydroxyl-α-sanshool under the same determination conditions. According to the mass spectrometry information combined with software analysis, the substance can be determined as hydroxyl-ε-sanshool. Obviously, hydroxyl-α-sanshool isomerized to produce hydroxyl-ε-sanshool with two homeopathic conjugated double bonds, and its transformation path is shown in Fig. 3(c).

The molecular weight of the peak 3 compound was 263 Da can be seen from Fig. 3(d). According to MS^2^ mass spectrogram and retention time of comparison standard substance, the substance was identified as hydroxyl-αsanshool. The sources of hydroxyl-α-sanshool are divided into two parts (Fig. 3(d)): One part is from a small amount of hydroxyl-α-sanshool that has not been completely transformed in the solution, part of hydroxyl-α-sanshool is transformed from hydroxyl-β-sanshool. The results showed that the two kinds of sanshools could be transformed into each other under certain conditions, which is consistent with the results of Cheng et al. (2021).

According to the mass spectrometry (Fig. 3(e)), the molecular weight of peak 4 compound was 263 Da. According to MS^2^ mass spectrometry and retention time, the substance can be identified as hydroxyl-β-sanshool. It has been stated previously that hydroxyl-β-sanshool can be transformed from hydroxyl-α-sanshool, which further proved that: Under certain conditions (including oxygen, light and acidic environment), hydroxyl-α-sanshool can be converted into isomers (Schweiggert et al., 2006, Topuz and Ozdemir, 2004, Yan et al., 2017). It is worth noting that the conversion rates of the two kinds of sanshool are not equal. From the slope of the first-order function simulation equation of sanshool degradation rate and concentration, it can be seen that the degradation conversion rate of hydroxyl-α-sanshool is higher than that of hydroxyl-β-sanshool.

The molecular weight of peak 5 compound was also 263 Da, indicating that the substance is the isomer of hydroxyl-α-sanshool (M = 263 Da) and hydroxyl-β-sanshool (M = 263 Da). According to MS^2^ mass spectrometry (Fig. 3(f)) and references, which can be inferred that the substance is:(1Z,2E,4E,8E,10E)-n-(2-hydroxy-2-methylpropyl)dodeca-2,4,8,10-tetraenimidic acid. And the specific transformation path (Fig. 3(f)) is as follows: First, hydroxyl-α-sanshool was converted to hydroxyl-β-sanshool, and then the carbonyl bond of hydroxyl-β-sanshool was broken, which leads to isomerization and isomer formation under the action of hydrochloric acid: (1Z,2E,4E,8E,10E)-n-(2-hydroxy-2-methylpropyl)dodeca-2,4,8,10-tetraenimidic acid.

**Fig. 4:**
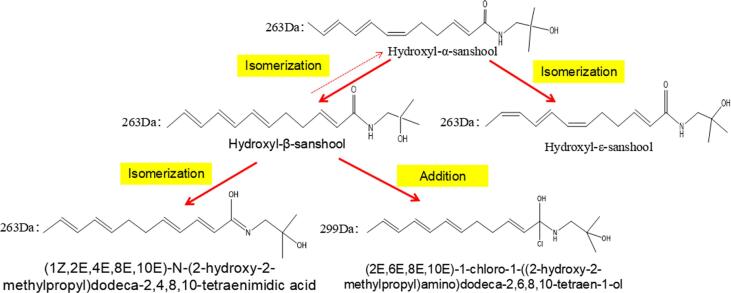
Diagram of transform path of sanshool exposed to acid environment.

In acidic environment, the content and grade of numbing substances decreased, which was not the degradation of numbing substances, but the transformation of numbing substances. And the transformation path of numbing substances can be divided into two steps (Fig. 4). Step 1: hydroxyl-α-sanshool isomerization under acidic environment, the product is hydroxyl-β-sanshool and hydroxyl-ε-sanshool, in this process, a small amount of hydroxyl-β-sanshool will also be converted into hydroxyl-α-sanshool. Step 2: Further isomerization of hydroxyl-β-sanshool produces isomerism: (1Z,2E,4E,8E,10E)-N-(2-hydroxy-2-methylpropyl)dodeca-2,4,8, 10-Tetraenimidic acid, and part of the hydroxyl-β-sanshool addition reaction with hydrochloric acid, the product was (2E,6E,8E,10E)-1-chloro-1-((2-hydroxy-2-methylpropyl)amino)dodeca-2,6,8, 10-Tetraen-1-ol.

## 4 Conclusion

In this work, ZBM was used as raw material, and orthogonal experiment was conducted on the basis of single factor experiment. The optimal degradation conditions of numbing substances were as follows: material size 0.180 mm, hydrochloric acid concentration 14%, temperature 35 °C and time 35 min. Under these conditions, DS1 value reached 75.56 ± 1.04%, DS2 value reached 42.69 ± 0.73%, and G value decreased from 5.22 ± 0.66 to 2.17 ± 0.91. The degradation effect of numbing substances was significate. In addition, HPLC, FT-IR and LC-MS were used to explore the principle for the degradation of numbing substances. The results showed that the first-order reaction rate equation of degradation rate and concentration of hydroxyl-α-sanshool and hydroxyl-β-sanshool were: S_1_ = 274.93–54.68e^0.1067c1^, S_2_ = 156.175–5.323e^0.2281C2^. When the concentration of hydrochloric acid reached 14%, the content of sanshools decreased 80% after 0.5 h. Which is the same reason capsaicin in chili peppers is weak in acidic environments (You & Cao, 2010). In acidic environment, under the influence of [H^+^], the flavoring groups of sanshools were affected, and the isomerization and addition reactions of sanshools occurred. The products of isomerization are hydroxyl-ε-sanshool and (1Z,2E,4E,8E,10E)-N-(2-hydroxy-2-methylpropyl)dodeca-2,4,8,10-tetraenimidic acid; The product of the addition reaction is (2E,6E,8E,10E)-1-chloro-1-(2-hydroxy-2-methylpropyl)amino)dodeca-2,6,8,10-tetraen-1-ol. In conclusion, the content of numbing substances decreased rapidly in acidic environment, which was mainly caused by isomerization and addition reactions of sanshools, which provided reference for numbing substances change of *Zanthoxylum bungeanum* and its products during storage, meanwhile, promoted the comprehensive utilization of ZBM.

## Declaration of Competing Interest

The authors declare that they have no known competing financial interests or personal relationships that could have appeared to influence the work reported in this paper.
