# Supplementary figures and images for: Degradation and transformation mechanisms of numbing substances: Hydroxyl-α-sanshool & hydroxyl-β-sanshool from Zanthoxylum bungeanum exposed to acid environment
Source: Food Chem X. 2022 May 21;14:100342. doi: 10.1016/j.fochx.2022.100342 (PMC9142844; doi:10.1016/j.fochx.2022.100342)

**Fig. 4** Diagram of transform path of sanshool exposed to acid environment.


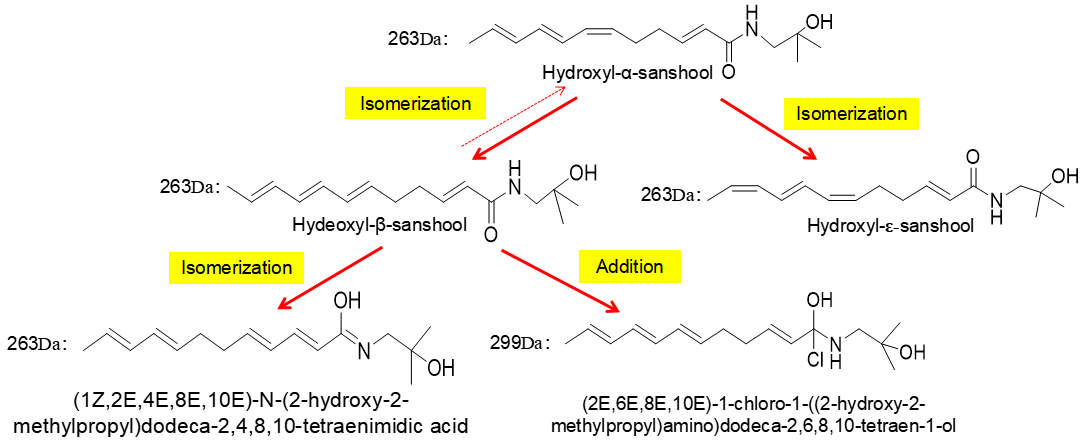


**Fig. 4**

Supplement: Supplementary data 1 [file mmc1.docx]
